# Supplementary material for: Are carbon emissions trading and green financial instruments synergistic? -Comprehensive quantitative research based on content analysis
Source: PLoS One. 2024 Mar 7;19(3):e0298601. doi: 10.1371/journal.pone.0298601 (PMC10919601; doi:10.1371/journal.pone.0298601)
Supplement: S1 Data — (ZIP) [file pone.0298601.s001.zip › Data/地级市Z_F工作报告.zip!/╔╜╬≈/╜·│╟╩╨/└┤╫╘╣1⁄2╓┌║┼í╛┬φ┐╦╩2╛▌═°í┐_2011.docx]

各位代表：

现在，我代表市人民政府向大会作工作报告，请予审议，并请市政协委员和其他列席人员提出意见。

一、2010年工作回顾

过去的一年，在省委、省政府和市委的正确领导下，在市人大及其常委会和市政协的监督支持下，市政府团结依靠全市人民，深入贯彻落实科学发展观，加快转变经济发展方式，着力推进产业结构调整，全面提高经济增长的速度、质量和效益，全市经济社会持续平稳较快发展。全年生产总值完成730.5亿元，同比增长13.7%；规模以上工业增加值完成435.7亿元，增长19.3%；全社会固定资产投资完成432.5亿元，增长22.2%；财政总收入完成153.2亿元，增长12.6%；一般预算收入完成55.5亿元，增长15.4%；外贸进出口总额完成5.3亿美元，增长120%；社会消费品零售总额完成191.1亿元，增长18.5%；城镇居民人均可支配收入完成17353元，增长14.5%；农民人均纯收入完成5899元，增长12.2%。市五届人大七次会议确定的各项工作任务和“十一五”规划的主要目标全面完成。

一年来，我们重点抓了以下几项工作：

——加快转变经济发展方式，结构调整扎实推进。一是开工建设了一批重大项目。153项重点工程完成投资313.7亿元，占年度目标的164%。100项工业转型项目有60项建成投产、33项开工建设；20项重点工业项目有12项建成投产、6项开工建设。二是基本完成了煤炭资源整合重组任务。协议签订率完成100%，换证率99.2％，接管到位率100%，资金补偿到位率83%。三是改造提升传统产业。煤炭产业着力提升煤炭开采的规模化、机械化、信息化水平，新规划了玉溪、东大等一批大型现代化矿井，为可持续发展奠定了坚实基础。电力产业稳步发展，国投热电一期工程完成，晋煤能源、华能阳城煤矸石电厂加快推进，阳城500千伏输变电工程竣工运行。煤化工产业在形成351万吨大颗粒尿素、111万吨甲醇、10万吨二甲醚、10万吨合成油的生产能力的基础上，发展重点转向现代煤化工。装备制造业重点发展精密铸造、煤机成套设备、防爆设备和汽车零部件等产品，朝着精密化、标准化、规模化方向发展。“一区六园”已见雏形，产业集聚程度提高。四是精心培育新兴产业。煤层气产业初步构建了抽采、液化、发电、管输、工业、民用等方面的开发利用体系，形成了年抽采30亿立方米的能力。高新技术产业加快推进，富士康科技工业园A区项目启动实施，高平唐一锂离子电池项目开工建设，支持乐百利特LED、路宝铝轮毂等产品做大做强。新材料、生物制药、电子信息等产业发展壮大。五是大力发展现代服务业。凤展国际广场、豪德光彩贸易广场、兰花国际物流园区、国贸中心、白马寺接待中心等一批服务业项目正在兴建，一批知名品牌入驻晋城。浦发、兴业、招商等商业银行在晋城设立分支机构。加强旅游基础设施建设，加大旅游产品营销力度，成功举办了“旅游文化月”活动。全年接待旅游人数增长19.5%，旅游总收入增长20%。

——着力强化“三农”工作，农村经济稳步发展。粮食总产量首次突破18亿斤大关，创历史新高。畜牧业生产稳定增长，生猪存、出栏分别增长11.8%和14.6%。蚕桑、果品、蔬菜、小杂粮、中药材等特色农产品基地建设力度加大，成为农民增收的新亮点。农村“五个全覆盖”任务提前完成。13.8万农户用上了沼气和秸秆气可再生能源，13个乡镇、78个村用上了煤层气。强力推进新农村建设和扶贫攻坚，150个贫困村的扶贫工作取得阶段性成效。

——全力推进特色城镇化，城乡建设成效显著。阳翼和西北环城高速公路竣工通车，高平至陵川高速公路具备通车条件。10项城市建设重点工程竣工完成。市区新增管道煤层气用户1.54万户，累计达到9.98万户。煤层气综合利用工程荣获“中国人居环境范例奖”和“迪拜国际改善居住环境最佳范例奖”。召开了全市特色城镇化建设沁水、高平现场会，7个城乡一体化发展示范片区有序推进。全市城镇化率达到48.4%，较上年提高1.4个百分点。

——加强节能减排和环保工作，生态环境持续改善。深入实施“蓝天碧水工程”，大力开展环境综合整治，推进化工行业废水超低排放技术改造，丹河人工湿地一期工程发挥效益，二期工程开工建设。6个县（市、区）环境空气质量稳定达到国家二级标准。市区空气质量二级以上天数达到354天，荣获第六届中华宝钢城镇环境类优秀奖。强力推进节能减排，加快淘汰落后产能，“十一五”约束性指标全面完成。实施了十大林业生态重点工程，启动了环城高速公路绿化，完成造林面积15万亩。

——继续深化改革开放，招商引资力度加大。继续深化国有企业改革，扎实推进事业单位分类改革，全面推进集体林权制度、文化、医药卫生、投融资体制改革。组团参加了国内重要展会，成功举办了“世界新兴产业合作论坛”和“晋城产业项目香港合作推介会”，实际利用外来资金139亿元。积极开展对外友好交往，启动海关、出入境检验检疫机构大楼建设。进一步创优民营经济发展环境，中小企业活力进一步增强。

——切实保障和改善民生，社会大局和谐稳定。科技、教育、人口计生、卫生、文化、体育等各项社会事业全面进步，国家创业型城市试点工作顺利推进，社会保障覆盖面和标准继续提高，为民办的16件实事全部兑现。安全生产保持了总体平稳的良好态势，事故起数、死亡人数、煤炭百万吨死亡率大幅低于省控目标。创新社会管理机制，深入开展“严打”整治斗争，强化信访和社会矛盾排查调处工作，“平安晋城”建设成效显著。坚持依法行政，着力打造“阳光”政府，深入推进精神文明和民主法制建设。自觉接受人大、政协监督，认真听取各民主党派、工商联和无党派人士意见。全年共办理人大代表建议246件、政协提案251件。民族宗教、妇女儿童、档案史志、外事侨务、文物保护、人防、气象、统计、地震、老龄、残疾人、红十字、慈善救助等社会事业都取得了新进展和新成绩。

各位代表，过去一年全市经济社会发展的成就来之不易。这是坚决贯彻执行党中央、国务院和省委、省政府一系列方针政策的结果，是中共晋城市委统揽全局、正确领导的结果，是市人大、市政协大力支持、有效监督的结果，是全市人民辛勤工作、共同努力的结果。在这里，我代表市人民政府，向全市人民，向驻市解放军指战员、武警官兵和公安干警，向各民主党派、工商联、各人民团体和社会各界人士，向所有关心和支持晋城发展的同志们、朋友们，表示衷心的感谢和崇高的敬意！

在总结成绩的同时，我们也必须清醒地认识到：经济增长过度依赖煤炭的状况没有根本性改变，支柱产业多元化的格局远未形成。支撑“十二五”发展的大项目、新项目不多，转型跨越发展的任务十分艰巨。环境容量约束增大，节能减排形势严峻。社会就业矛盾突出，城乡居民增收困难。部分干部群众安于现状，加快转型跨越发展的紧迫感不强。站在又一个五年规划的新起点，我们必须只争朝夕，锐意进取，决不能使结构调整这个永恒的主题变成永远的难题，决不能把转型发展这个紧迫的课题当作遥远的话题，扑下身子，真抓实干，下决心走出一条符合晋城实际的宽广的科学发展之路。

二、2011年工作安排

2011年是“十二五”规划的起步年，是加快发展方式转变、开辟转型跨越发展新局面的基础年，我们还将迎来中国共产党成立90周年。做好今年的政府工作，意义更加深远，责任更加重大。

今年政府工作的总体要求是：全面贯彻党的十七大和十七届三中、四中、五中全会精神，在中共晋城市委的正确领导下，坚持以科学发展观为主题，以转变经济发展方式为主线，以推进国家资源型经济转型综合配套改革试验区建设为统揽，以当好全省以煤为基、多元发展先行区示范区为目标，大力推进工业新型化、农业现代化、市域城镇化、城乡生态化，深化体制机制改革，扩大对外对内开放，加快推进社会事业全面进步和民生进一步改善，在实现转型跨越发展上迈出坚实的步伐。

全市经济社会发展的主要预期目标是：地区生产总值增长13%以上，全社会固定资产投资增长22%，社会消费品零售总额增长16%，财政总收入、一般预算收入分别增长15%，城镇居民人均可支配收入增长12%，农民人均纯收入增长12%以上，居民消费价格总水平涨幅控制在4%左右，城镇登记失业率控制在4.2%以内，新增城镇就业岗位2.38万个。

约束性指标是：万元GDP综合能耗和二氧化碳排放量分别下降3.5%、3.7%，万元工业增加值用水量下降4.4%；二氧化硫、化学需氧量分别下降2.36%、2%，氮氧化物、氨氮排放量分别下降2.19%、1.83%，烟尘、粉尘排放量分别下降2%。

实现上述目标，做好政府工作，必须把科学发展观作为推动工作的行动指南，自觉地用科学发展的理念想问题、做决策、办事情，创造性地开展工作；必须正视经济增长过度依赖煤炭这一现状，切实增强转型发展的现实紧迫感和历史责任感，积极培育战略性新兴产业，努力实现支柱产业多元化；必须紧紧抓住国家实施山西省资源型经济转型综改试验区和建设中原经济区的战略机遇，用更加开阔的视野、在更大的空间中挖掘晋城的优势，谋划晋城的发展；必须坚持解放思想、改革开放，不断破除体制机制障碍，把激活内力与借助外力结合起来，为经济社会发展提供强大的动力支撑；必须坚持工业化、城镇化、生态化一同推进，城乡一体化一同推进，市域经济与县域经济一同推进，拓展区域统筹协调发展的新境界；必须把改善民生作为经济社会发展的最高目标，切实解决好人民群众最关心、最直接、最现实的利益问题，让人民群众得到的实惠程度、对政府工作的满意程度与经济社会的发展程度同步提高。

今年，要重点抓好以下八方面工作：

（一）加快推进产业结构优化升级，着力增创经济发展新优势。

加快传统产业新型化。煤炭产业要深入推进煤炭资源整合煤矿兼并重组，提高煤炭洗选率和加工转化率，实现煤炭行业的高效、绿色、安全发展。加快68个整合改造项目的建设进度，年内完成高平前和、泽州坤达等13座矿井的联合试运转或竣工投产，新增有效产能1000万吨；推动煤炭行业积极发展煤炭物流、煤机装备制造等相关产业，不断提高非煤产业比重，加快培育现代煤炭企业集团。电力产业要运用先进的节能环保技术进行新型化改造，重点抓好晋煤能源公司2×13.5万千瓦煤矸石发电的投产，积极推进高平煤电一体化、华能阳城电厂等项目的前期工作。同时，要超前谋划电网规划，加快电网建设步伐。今年完成阳城北500千伏输电站工程，建设泽州东、沁水郑庄、阳城凤城220千伏增容和城区北石店、泽州水东等9项110千伏增容改造工程，实施城乡电网改造升级工程。化工产业，要加大力度调整化肥企业的产品方向，延伸产业链，增加附加值。鼓励推动化肥企业与煤炭企业的重组整合，抓好兰花科创30万吨硝基复合肥、7万吨己二酸、5万吨三聚氰胺项目。

推进新兴产业规模化。在煤层气产业方面，要进一步加大勘探力度，摸清资源的储量、分布特点，开展促进产业发展的高层论证与政策研究，争取国家对煤层气产业发展的新政策。加强与煤层气开发企业的合作，携手推动煤层气开发利用的规模化、产业化。加大对煤层气产业的规划，拓宽煤层气应用领域，提高煤层气就地转化率、液化率。加快兰花煤层气田的开发。启动实施城乡居民生活用气全覆盖工程，打造全国煤层气之都，为“气化山西”做贡献。在现代煤化工产业方面，要加快推进晋煤集团煤化工循环经济园区年产2×60万吨烯烃、100万吨清洁燃料项目和兰花集团年产6万吨聚甲醛项目建设。推进兰花集团与中石化合作180万吨甲醇60万吨烯烃、国投180万吨甲醇60万吨烯烃项目的前期工作。在新能源产业方面，要大力扶持锂离子电池、太阳能光伏等产业，推进唐一锂离子动力电池、中道能源锂离子电池、融高太阳能光伏电池、兰花单晶硅多晶硅及电池组件、硕阳光电太阳能单晶硅项目建设。在高新技术产业方面，要积极发展电子信息、生物制药、节能环保等产业，重点抓好富士康A区、晋煤乐百利特大功率LED芯片及节能灯具、裕丰碳酸钙新材料等项目建设。特别要把富士康A区建设作为一个转型发展的战略性项目，坚定不移地推进。在冶铸和装备制造业方面，要加快整合重组、上档升级、延伸拓展，促进产业向集群化方向发展。着力抓好金鼎煤机、清慧汽车配件、天巨重工、路宝铝轮毂二期、兰花汉斯瓦斯抑爆二期等项目。特别是要采取更加有力的措施，推动铸管件、汽车配件、煤机制造三个行业的发展。在轻纺工业方面，要整合丝麻产业资源要素，抓好绿洲大麻二期、佶利尔异地改扩建工程和蚕桑、丝麻基地建设，做大企业规模，做强产品品牌。全力支持兰花集团进一步做大做强，使其成为具有更强竞争力和影响力的现代煤炭与煤化工企业集团。

增强园区产业集聚功能。园区是项目建设的载体，是招商引资的平台。要加快推进晋城经济技术开发区、高平煤电化工业园、泽州巴公装备制造工业园、北留周村煤电化工业园、南村新兴产业工业园、阳城芹池现代煤化工工业园和沁水新能源产业工业园的建设。要根据区域自然环境、资源条件和产业方向，搞好园区规划建设。提高引进项目的质量，充分发挥集聚效应，加快企业向园区集中，项目向园区布局，产业在园区提升，政策向园区倾斜、人才向园区流动，构建“设施齐全、功能配套、布局合理、特色突显、资源共享、产业循环、服务优质、生态良好”的工业园区。

大力发展以旅游业为龙头的现代服务业。加大旅游景区建设和产品营销的力度，注重文化与旅游的互动融合，打造沁河古堡、太行云顶、城郊休闲、蟒河山水、历山生态、丹河文化六大龙头景区，丰富山水休闲度假、历史文化体验、红色旅游、农家乐休闲、工业旅游五类旅游产品，建立旅游集散、购物、会展、演艺、接待五大配套服务体系，构建全市旅游“精品线路”。加快豪德、兰花、晋运等几个大型物流园区和配送中心的建设，发展金融保险、现代物流、信息咨询等生产性服务业。打造煤层气开发企业区域总部基地。兴建一批大型购物中心、星级酒店、品牌服饰专卖店等，推动传统服务业的上档升级，充分发挥生活性服务业在扩大消费中的重要作用，打造山西东南部乃至中原城市圈具有较强辐射力的区域性商贸物流中心、生态旅游文化中心和休闲娱乐度假中心。

抓好项目建设工作。必须把项目建设作为推动转型跨越的第一抓手，统筹推进各个领域、各个方面的重大项目建设。重点抓好169项重点工程、100项工业转型项目和30项重点工业项目、100项重点新建技改项目、50项自主研发项目和500项创业项目建设。要促进金融与产业间的结合，加强金融手段的应用，推进金融行业参与全市各类重大项目的前期论证，加强政府对银企关系的协调，建立重点企业对银行的资金需求直报制度，真正形成“政府搭台，企业唱戏，金融弹琴”的项目共推机制。要建立项目建设的创意策划储备机制，设立“三个一同推进、创意策划晋城”奖励基金，鼓励调动更多、更大范围的社会力量，聘请国内外一流的产业策划团队，谋划晋城、设计晋城、发展晋城。

各位代表，转变经济发展方式，既要以煤为基，更要多元发展。要以登高望远的境界，以“不求有功于我、但愿庇荫后人”的历史责任感、使命感，勇于担当，奋力推进经济结构战略性调整！

（二）大力发展现代农业，推动“三农”工作迈上新台阶。

进一步落实强农惠农政策。在认真落实好国家、省一系列强农惠农政策的基础上，建立完善我市财政支农的政策体系，进一步加大各级财政对“三农”的投入。着力巩固以工补农、以城带乡的阶段性成果，健全可持续发展的机制体制。充分发挥政策调控作用，引导民间资金投向现代农业，实现农村经济稳步发展。

加强农业基础设施建设。认真落实中央1号文件精神，大力发展民生水利，抓好新水源工程建设，建设小型水库31座，除险加固重点小型水库11座，新建和改造末级渠系300公里。完成中低产田改造10万亩。加强农机化新技术推广，提高农业机械化水平。

大力发展现代农业。严格保护耕地，确保基本农田不减少，稳定粮食生产。积极引进推广国内外优良品种和先进技术，抓好高效农业科技示范园和现代农业示范基地建设。优化农业产业布局，做强做大做优畜牧、蚕桑、小杂粮、中药材、丝麻、蔬菜等特色产业。启动实施“一村一品”百村示范、百村推进工程，培育扶持特色农业示范基地县。积极推进农业产业化，通过“公司+合作社+基地”的模式，提升农产品深加工的规模和水平。整合我市名优农产品牌，立足打造“晋城？中国南太行”绿色、有机地理农产品牌与国内外知名连锁超市的对接，提升我市农产品的市场影响力。

千方百计确保农民收入大幅增长。整合农业、教育、劳动等部门资源，加大农民培训和转移就业力度，严格执行最低工资保障制度，促进农民工资性收入大幅增长；全力组织实施农业农村工作十大重点工程，加大对设施蔬菜、“513”龙头企业、标准化养殖、干鲜果经济林建设的投入力度，建立扶持农民多种经营、自主创业的体制机制，促进农民家庭经营性收入快速增长；稳步推进土地承包经营权流转，切实加强农村资金、资产、资源管理，认真落实各项支农惠农政策，促进农民财产性和转移性收入持续增加。多措并举，确保农民人均纯收入增幅达到12%以上，力争有大的突破，为“十二五”农民收入翻番奠定坚实基础。

扎实推进扶贫攻坚和新农村建设。加大陵川、沁水两县片区扶贫开发规划、扶贫项目推进力度。市财政扶贫资金提高到2500万元。抓好130个省级重点推进村的新农村建设。要在完善提升原有“五个全覆盖”工程的基础上，启动实施农村中等职业教育免费、新型农村社会养老保险、农村街巷硬化、便民连锁商店、农村文化体育场所新的“五个全覆盖”工程，力争在全省提前一年完成农村中等职业教育免费、新型农村社会养老保险两个全覆盖。

各位代表，做好“三农”工作，事关经济社会发展大局。我们要把解决好“三农”问题作为重中之重，摆在突出的位置，在工业化、城镇化深入发展中同步推进农业现代化，促进农村经济社会持续、健康、快速发展，使广大农民群众得到的实惠更多、更快、更好！

（三）加快推进特色城镇化建设，拓展城乡统筹发展新境界。

工业化需要城镇化来承载，现代化需要城镇化来表现。要把城镇化作为统筹城乡发展的重要抓手，作为转型跨越发展的重要支撑，加快建设步伐，确保今年全市城镇化率达到50.2%。

进一步完善城乡规划体系，强化规划的引领力。按照全省“一核一圈三群”的城镇化战略和我市“六区联动、组团发展”的城市空间布局，实现中心城市控制性详细规划全覆盖，进一步完善各类专项规划。研究制定全市城镇化推进实施方案和相关政策措施，编制“一城两翼”城镇组群发展规划和沁河古村镇保护与发展规划，配合做好晋东南城镇群规划纲要编制工作。各县（市、区）要相应做好县域城镇体系规划、中心村镇规划修编、历史文化名镇名村保护规划和风景名胜区总体规划大纲编制等工作。发挥规划的引领作用，在城镇化建设中充分体现“方便、温馨、开放、现代”的城市形态和文化格调。

继续推进基础设施建设，增强城镇的通达性、功能性。以创建国家卫生城市、国家森林城市、全国文明城市为目标，进一步完善城市功能，提升城市品位，增强城市的吸纳力、影响力和承载力。在公路交通方面，建成高陵高速公路陵川至省界段，加快推进高沁高速公路建设，完成阳城至济源、安泽至郑庄高速公路前期工作。加快建设晋城至高平、晋城至阳城、环城高速至晋济高速连接线等三条一级公路。完成市汽车客运东站建设工程，开工建设苗匠、北石店公交场站和高平、阳城二级汽车客运站。在铁路建设方面，协助做好太焦高速铁路前期工作，争取晋城至焦作城际高铁早日开工。继续推进嘉南铁路建设。在城市建设方面，推进畅安路、兰花北路、凤城南路、金村大道、红星东街和红星西街延伸段6条城市道路工程建设，启动陵沁线改线及主城区到金村区的东西向主干道建设前期工作，促进北石店、南村、金村新区的发展。完善城市热网工程。提升北石店区城市化水平，加快20个城中村改造步伐。继续扩大民用煤层气覆盖面，四个县城覆盖率要达到50%以上，新增用户3万户。

加快推进特色城镇化建设，实现中心城市、县城、城镇、新农村的互促互动。深入实施“中心城市、县城、小城镇、新农村”四位一体发展战略，提高城镇建设的质量和水平。中心城市要以扩容提质为重点，改旧城、保古建、建新区，提升建设水平、管理层次和文明程度，增强综合承载和辐射带动能力。县城要加快实施垃圾无害化处理场建设，大力推进集中供热、园林绿化、污水处理、燃气、道路、供水工程建设，不断完善县城的城市功能。中心镇要以工业化带动城镇化、以城镇化助推工业化，全面提高小城镇集聚能力，形成各自的产业特色、文化特色、景观特色，建设一批经济强镇、商贸重镇、旅游要镇、历史名镇和园林新镇。重点抓好沁河沿线、丹河沿线、环城高速周边的城乡一体化示范片区建设。研究制定城乡一体化考核试行标准，总结经验、抓好典型，带动全市城镇化建设。

创新加快城镇化进程的体制机制。加快推进户籍制度改革，以多种途径放宽城镇户籍限制，让符合条件的农业人口有序变为市民、融入城镇。完善城乡就业和社会保障制度，实现城乡劳动力市场一体化、农民工与城镇产业工人劳动待遇平等化和社会保障全覆盖，农民工子女城市就学与城镇居民子女同等待遇。

（四）加大节能减排和环境保护力度，提升生态文明建设新水平。

狠抓节能减排工作。突出抓好工业领域节能，继续淘汰落后产能，运用先进技术改造提升传统产业的装备水平，加强新上项目能评工作，实施重点节能项目50项。大力推进建筑、交通运输等领域的节能工作。强化全民节能意识，积极倡导绿色消费。强力推进污染减排，严格“两高一资”项目的环境准入标准，大力推进规划环评，构建全方位的污染防控体系，四个县级污水处理厂实现稳定正常运行。

加大环保整治力度。持续推进“蓝天碧水工程”， 全面加大区域环境综合整治，推动大气环境质量持续改善提升，县（市、区）环境空气质量继续稳定达到国家二级标准。加大对沁河的保护力度，完成丹河人工湿地二期工程，丹河、沁河出境断面达到功能类别要求，全市集中式饮用水源地水质达标率保持100%，保障乡镇、农村饮用水安全。整体推进农村环境保护，改善农村生产生活条件。

加强生态林业建设。大力实施生态立市战略，坚持“节约型、多功能、园林式”的绿化理念。围绕创建国家森林城市，抓好城市生态园林绿化，完成新植物园、泽州公园二期改造工程，开工建设龙湾公园，改造提升赵树理公园。继续深化林权制度改革，加快推进造林绿化、森林资源管护和林业产业开发。全年完成荒山造林15万亩、通道绿化122公里、巩固建设10个名优干果经济林乡镇、新建100个林业生态村、新建5万亩干果经济林基地。要在全社会倡导“树木树人”、“树木敬人”的风尚，建立各种各样的团体纪念林、家庭纪念林和社会公益林，真正使植树成为家庭的自觉，成为社会的情怀！

大力发展循环经济。要以国家级循环经济标准化试点城市建设为契机，大力发展清洁生产、节能技术和节能环保服务、环保装备、环保产品，使循环经济成为基本经济形态、产业发展模式和新的竞争优势。按照“多联产、全循环、抓高端”的思路，规划、建设、改造各类工业园区，全面推进支柱产业特别是传统支柱产业的循环化发展，延伸资源循环产业链，提高资源综合利用水平，实现发展效益最大化。

各位代表，蔚蓝的天空、秀丽的山川、葱茏的森林、园林的城市，是晋城市的品牌，是晋城人的自豪。珍视生态，就是珍视我们的生命；爱护生态，就是爱护我们的子孙；建设生态，就是建设我们的家园。在加快工业化、城镇化的进程中，我们一定要加强资源节约型、环境友好型社会建设，把一个更加良好的生态环境留给子孙后代！

（五）加大改革开放力度，增添经济社会发展新活力。

适应社会主义市场经济建设的新要求，推进体制机制创新，扩大对外对内开放，用市场的力量把劳动、知识、资本、资源、管理等生产要素配置得更加优化，让经济社会发展内在活力释放得更加充分。

深化体制机制改革。加快国有和集体企业改革改制，完善企业内部法人治理结构，鼓励企业进行战略重组整合。大力发展资本市场，支持企业直接融资，积极培育上市公司。深化乡镇机构改革，积极稳妥推进事业单位分类改革。全面推进医疗卫生体制改革五项任务的落实。继续深化文化体制改革。通过改革，为推动经济社会发展提供制度保障和动力源泉。

扩大对外开放力度。要进一步转变思想观念，营造开放氛围，让开放成为我们的思维方式，让开放成为我们的工作方法。要加大招商引资力度，建立重点项目领导负责制、县级政府目标责任制、部门的服务承诺制和项目策划、项目储备、对外联络、招商目的地跟踪、责任主体考核奖惩机制，设立招商引资中介人才库和项目建设学习制度。要创新招商引资方式，重点推进区域性定向招商、产业性专业招商、龙头企业带动集群化招商等模式，吸引国内外大企业、大集团、重点民营企业和高层次人才来投资兴业。积极参加大型展会和贸易洽谈活动，重点做好第六届中博会、渝洽会、投洽会的参展工作。举办好“中国？晋城太行山国际旅游文化节”，组织好赴上海、韩国的招商引资活动。加强与中央和省属大企业的合作，支持他们在晋城投资发展。特别要大力支持晋煤集团、煤运集团在晋城的多元发展，充分发挥他们在加快我市产业结构调整和城市化进程中的作用。加强对外交流合作，促进与周边城市间互动发展，以煤为基、立足山西，多元发展、融入中原。

大力发展民营经济。民营经济和中小企业是推动转型跨越发展的重要力量、活跃力量。要进一步完善加快民营经济发展的政策措施，充分利用我市民间资本充裕的优势，大力支持、鼓励、引导民间资本向非煤产业转移、参与国有企业的改制重组，参与城市基础设施建设和社会公益事业。要强化服务，帮助民营企业拓宽融资渠道，解决中小企业融资难问题。完善中小企业管理培训、质量检验、信息服务等社会化服务体系。引导和支持民营企业开展技术创新，培育具有自主知识产权的品牌。鼓励民营企业与行业龙头企业间的合作发展，培养出我们自己在国内同行业有影响力的龙头企业。上半年召开全市发展壮大民营经济推进大会，在全社会营造创业光荣的氛围，尊重民营企业家的劳动价值，尊重民营企业家的社会贡献。

提高自主创新能力。以加快建设创新型城市为目标，建立和完善多元化科技投入体系。重点围绕改造提升传统产业，培育壮大新兴产业等核心技术进行科技攻关。建立产学研相结合的多层次、多形式的科技协作。抓好高层次创新型科技人才、新兴产业创业型领军人才、重点领域急需紧缺人才的培养、引进和开发，为加快转变经济发展方式提供智力支撑。

　　积极抓好“十二五”规划编制完善和“转型综改试验区”相关工作。要放宽视野，精心谋划，科学论证，高标准、高质量完成“十二五”规划编制任务。做好国家资源型经济转型综合配套改革试验区的相关工作，抢抓政策机遇，抓紧制定并落实好各项实施方案，先行先试，大胆探索，力争在一些领域有突破性进展。

（六）立足保障改善民生，促进各项社会事业新发展。

更加注重扩大社会就业。坚持把就业作为经济社会发展的优先目标。围绕创建国家级创业型城市，重点抓好创业孵化基地建设。采取免费培训、减免税费、提供小额贷款等措施，大力扶持自主创业和自谋职业，以创业带动就业。多渠道开发就业岗位，做好高校毕业生、零就业家庭、就业困难人员的就业工作。完成城镇新增就业2.38万人，创业带动就业8000人，转移农村劳动力1.83万人，失业人员再就业5500人，困难人员就业1400人。

更加注重社会保障体系建设。扩大城镇职工养老、医疗、工伤、失业保险参保范围。逐步提高企业退休人员养老金、城乡低保和五保供养标准。完善城镇职工、城镇居民基本医疗保险制度和新型农村合作医疗制度。加强社会救助体系建设，完善各类救助制度。抓好经济适用房、公租房、廉租房等保障性住房建设。贯彻落实房地产市场调控政策，稳定住房价格，推动房地产业健康发展，让城乡居民住得更宽敞、更明亮、更舒适。

更加注重优先发展教育。认真贯彻落实国家和山西省中长期教育改革发展规划纲要，优化提升教育资源布局。扎实推进义务教育均衡发展。加大教育投入，确保义务教育经费、“两免一补”和职业教育补助资金等落实到位，将农村中小学校生均公用经费补助标准分别由500元、300元提高到600元、400元。启动学前教育普及工程，抓好普通高中教育、民办教育、继续教育和特殊教育。大力发展职业教育，强化技能培训，培养高素质的劳动者，使高技术人才成为我市产业竞争新优势、新品牌。加强教师队伍建设，提高教育教学质量。抓好中小学校舍安全改造新建和设施配备工程。加快晋城学院建设和市中等专业学校的搬迁扩建工程，新建凤城中学、汇迁小学，启动秀水苑学校前期工作。

更加注重卫生、文化、体育等社会事业发展。完善基层医疗卫生服务体系，完成497个村级卫生室达标验收，在所有政府举办的基层医疗卫生机构实施国家基本药物制度，推进基本公共服务均等化。建立健全疾病预防控制体系，全面提升全市医疗卫生服务管理水平。加大创建国家卫生城市力度，完成创卫技术评估。全面启动晋城大医院项目的前期工作，建设一座设施一流、功能齐全、医技精湛，能够满足群众需求、辐射周边地区的现代化综合性医院。进一步稳定低生育水平，做好统筹解决人口问题各项工作，促进人口均衡发展。积极落实各项配套资金，推动公共文化服务体系的建设。扶持公益性文化事业，大力发展文化产业，满足人民群众日益增长的精神文化需求。尽早开工晋城大剧院建设，加快推进市美术馆、图书馆、档案馆“三馆合一”工程。加强文化遗产保护、利用和传承。大力开展全民健身活动，促进群众体育和竞技体育协调发展。

各位代表，民生就是人民的生存，民生就是人民的生活，民生就是人民的生命。我们要把保障和改善民生作为经济社会发展的最高目标孜孜以求，把更多的财力、物力投向民生，让人民群众生活得更加殷实、更加体面、更有尊严、更加幸福！

（七）加强社会主义民主法制和精神文明建设，开创和谐社会新局面。

积极推进依法治市进程，加强社会主义民主法制和精神文明建设，加强和创新社会管理，为经济社会发展提供有力保障。

加强社会主义民主法制建设。认真执行人大及其常委会的决议、决定，依法向人大及其常委会报告工作，自觉接受人大及其常委会的法律监督和工作监督。积极支持人民政协履行政治协商、民主监督、参政议政的职能。认真办理人大代表的建议、批评、意见和政协提案。广泛听取各民主党派、工商联、无党派人士和工会、共青团、妇联等人民团体的意见和建议。不断健全民主制度，丰富民主形式，拓宽民主渠道。完善基层民主自治和企事业单位民主管理制度，依法实行民主选举、民主决策、民主管理、民主监督。扩大政务公开、村务公开和厂务公开的范围和内容，保障人民的知情权、参与权、表达权、监督权。启动“六五”普法教育，加强和改进法律服务和法律援助工作，提高法治晋城建设水平。

加强精神文明建设。深入推进文明城市创建工作，提高全社会的现代文明素养。积极推进社会公德、职业道德、家庭美德、个人品德建设，充分发挥道德模范的示范作用。广泛开展群众性精神文明创建活动。加大城市形象的展示推介力度，提升晋城在全国的美誉度和影响力。

加强和创新社会管理。要完善社会管理机制，健全社区服务和管理网络，重视发挥群众组织、社会组织在社区管理中的积极作用。加强和改进信访工作，畅通群众诉求渠道，积极化解社会矛盾。全面加强应急管理，完善应急管理机制，积极预防和妥善处置自然灾害、事故灾难、公共卫生事件和社会安全事件等突发事件。健全社会治安防控体系，加强对重点人群、重点区域和重点组织的管理。推进“天网工程”和“平安社区”建设，依法严厉打击各类违法犯罪活动，增强群众安全感。进一步完善食品、药品安全监管体制机制，加强监管执法，提高食品、药品安全保障水平。加强防灾减灾体系建设。落实好民族宗教政策。加强统计、外事、侨务、对台、档案、史志、气象、地震、老龄、残疾人、红十字、慈善救助等工作。发展人民防空事业。继续支持国防和军队建设，做好双拥工作，积极促进军民融合式发展。

强化安全生产工作。进一步树立安全是发展的前提、安全是发展的保障的理念。把做好安全生产工作、保障人民群众生命财产安全作为重大政治责任。进一步强化企业安全生产主体责任落实，加快开展安全生产标准化建设和安全生产基层基础建设。进一步落实各级政府的安全监管责任，完善安全生产监管、应急救援、科技支撑三大体系。深入开展各行业各领域安全生产专项整治，突出抓好煤矿、非煤矿山、道路交通、危险化学品、特种设备、易燃易爆物品、森林防火和公共场所的安全管理。严格安全目标责任考核和安全事故责任追究，坚决遏制重特大事故发生，把安全生产各项指标严格控制在省定指标以内，确保全市安全生产形势持续稳定。

（八）加强政府自身建设，提高服务转型跨越发展新本领。

我们的政府是人民的政府，我们的权力来自人民，我们的天职是为人民服务。面对转型跨越发展的艰巨任务，面对经济社会发展的重重矛盾，面对人民群众的殷切期盼，政府各级公务人员必须进一步加强自身建设，切实做到在其位、谋其政、干其事、不误事、能成事、不出事。

加强学习，提高履职能力。要把学习作为一种工作态度和工作方式。坚持理论与实践相结合，也要坚持实践与理论相结合，提高分析问题、把握全局的能力和战略决策的水平。要注重国家政策的学习，提高掌握政策、运用政策、用足政策、用活政策的水平；注重社会管理知识、领导方法、领导艺术的学习，提高解决社会复杂矛盾的水平；注重现代科技知识、专业知识的学习，提高研判国际国内科技和产业发展最新动态的水平；特别要加强操作性市场经济知识的学习，提高运用市场的思路和办法、解决改革发展难题的水平。

依法行政，规范行政行为。进一步转变政府职能，增强服务意识，减少审批事项、简化审批手续，严格依照法定权限和程序履行职责、行使权力，提高行政效能。完善科学决策机制，健全重大行政决策听取意见、咨询论证、合法性审查、集体讨论和实施情况后评价制度。健全预防和化解行政争议机制，加强行政复议工作。

转变作风，狠抓工作落实。要大兴调查研究之风，围绕今年以及“十二五”工作重点，深入基层了解一手情况。各级领导既要当好战略家、设计师，又要当好战术家、工程师。克服只在指标思路上谋划工作，只在会议文件上落实任务的飘浮作风。要健全完善领导分工负责制、岗位责任制、奖惩考核制、首办负责制、限时办结制、行政不作为和过错责任追究等制度。强化各项重点工作落实的量化考核和监督监察，切实做到有布置、有检查、有考核、有奖惩。

严格监督，做到廉洁从政。推进惩治预防腐败体系建设，强化政府公务人员的廉洁自律意识。加强领导干部经济责任审计，完善政府重大投资项目公示制和责任追究制。加强对土地管理、工程建设、环境保护等重点领域的行政监察和审计监督，严肃查处各类违法违纪案件。牢固树立过紧日子的思想，减少不必要的行政开支，切实把有限的资金更多地用在经济社会发展和民生改善上。

在做好以上各项工作的同时，今年我们还要着力为人民群众办好以下10件实事：一是为100所农村中小学校配备体艺器材，为100所农村寄宿制学校增添卫生安全设施。二是再扶持贫困村220个，移民搬迁2000人。三是开工建设各类保障性住房13000套。四是建设设施蔬菜10000亩，使我市淡季蔬菜自给率提高到40%。五是市区新增公交车40台，新开公交线路3条。六是改造提升市级粮油配送中心和100家放心连锁店。七是建设新型农户储粮仓20000个。八是为城市社区配发康复训练器材，对农村200名贫困残疾人实施康复救助。九是硬化市区20条背街小巷路面。十是新发展100家星级“农家乐”。

各位代表，我们已经站在新的历史起点，迈上新的发展征程。让我们在中共晋城市委的坚强领导下，全面落实科学发展观，以更加饱满的热情、更加昂扬的斗志、更加扎实的工作，全力推进转型发展、跨越发展，真正把晋城建设成为“方便、温馨、开放、现代”的宜居之城、活力之城、幸福之城！
